# Pulmonary manifestations, treatments and outcomes of IgG4-related disease–a systematic literature review

**DOI:** 10.1007/s00296-024-05611-7

**Published:** 2024-05-20

**Authors:** Cristina Dragos, Clerin Joseph, Helen Elwell, Mrinalini Dey, Koushan Kouranloo

**Affiliations:** 1 Liverpool University NHS Foundation Trust, Prescot Street, Liverpool, L7 8XP UK; 2 British Medical Association Library, BMA House, Tavistock Square, London, WC1H 9JP UK; 3 Centre for Rheumatic Diseases, Weston Education Centre, Cutcombe Road, London, SE5 9RJ UK; 4 School of Medicine, Cedar House, University of Liverpool, Ashton Street, Liverpool, L69 3GE UK; 5 Department of Rheumatology, University Hospital Lewisham, High Street, Lewisham, London, SE13 6LH UK

**Keywords:** IgG4-related disease, Autoimmune disease, Lung disease, Comorbidity, Autoinflammatory disease

## Abstract

Immunoglobulin G4–related disease (IgG4-RD) is a multisystem fibroinflammatory condition. A consistent feature of many cases is pulmonary infiltrates, or respiratory failure. This systematic literature review aims to summarise the pulmonary manifestations of IgG4-RD, including clinical outcomes and treatment. This review was registered on PROSPERO (CRD42023416410). Medline, Embase and Cochrane databases were searched for articles discussing IgG4-RD syndrome. Information was extracted on demographics, type and prevalence of pulmonary manifestations, treatment and clinical outcomes. Initially, after deduplication, 3123 articles were retrieved with 18 ultimately included. A pooled total of 724 patients with IgG4-RD were included, 68.6% male, mean age 59.4 years (SD 5.8) at disease onset. The most frequently described pulmonary manifestation was mediastinal lymphadenopathy (n = 186, 48.8%), followed by pulmonary nodules (n = 151, 39.6%) and broncho-vascular thickening (n = 85, 22.3%). Where treatment was reported, the majority of patients received glucocorticoids (n = 211, 93.4%). Other immunosuppressive therapy included cyclophosphamide (n = 31), azathioprine (n = 18), with mycophenolate mofetil (n = 6), rituximab (n = 6), methotrexate (n = 5) and other unspecified immunomodulators (50). Clinical outcomes were reported in 263 patients, where 196 patients had remission of their disease, 20 had relapse, 35 had stable disease, four had progression and eight patients died from complications of IgG4-RD. This systematic review summarises pulmonary manifestations, treatments and outcomes in patients with IgG4-RD. Pulmonary involvement in IgG4-RD is relatively common, leading to high levels of morbidity and mortality. Glucocorticoids remain the mainstay of treatment, but further work is required to explore the management of patients with pulmonary manifestations in association with IgG4-RD.

**Supplementary Information:**

The online version contains supplementary material available at 10.1007/s00296-024-05611-7.

## Introduction

Immunoglobulin G4–related disease (IgG4-RD) is an autoimmune, fibroinflammatory condition, which may have single or multisystemic involvement, occurring in a chronological or metachronous fashion [1, 2]. IgG4-RD can present with clinical or radiological lesions in organs including the lungs, lymph nodes, salivary glands, lacrimal glands, liver, bile ducts and gallbladder, gastrointestinal system, retroperitoneum, skin, as well as the pancreas, in which it was first described [3, 4].

IgG4-RD is commonly characterised by raised serum levels of IgG4 (although this may not necessarily be present), dense lymphoplasmacytic infiltration containing IgG4-positive-plasma cells of the lesion and storiform fibrosis on histology, and good response to corticosteroid treatment [3, 5].

Pulmonary manifestations of IgG4-RD have been described, and can be highly variable, including pulmonary infiltrates, mediastinal lymphadenopathy, pleural disease, or pulmonary nodules, amongst others [2, 6]. These may be the first presentation of IgG4-RD, and may present alone, or in association with other organ involvement [7]. The symptomology can be equally wide-ranging and non-specific, including, cough, dyspnoea, fever and chest pain. IgG4-RD affecting the lungs can therefore mimic a variety of conditions including other immune-mediated conditions (e.g. vasculitis, sarcoidosis), infections and malignancies (e.g., lymphoma, lung cancer).

Comprehensive diagnostic criteria for IgG4-RD were established in 2011 and revised in 2019 [8]. Appropriate investigations and diagnostic work up are of paramount importance for patients with IgG4-RD presenting with pulmonary disease, especially due to the many (sometimes life-threatening) mimics. However, definitive diagnosis remains difficult to achieve, due to the rarity of IgG4-RD overall, its ambiguous presentation, and difficulties in obtaining tissue sample. This can lead to delays in treatment [9, 10], and, ultimately, long-term complications or potential unnecessary procedures on patients, leading to increased symptom burden and morbidity.

This systematic literature review (SLR) aims to summarise the clinical characteristics and prevalence of pulmonary manifestations in IgG4-RD, their treatment strategies, and outcomes, which may be used to aid clinicians managing patients with possible and confirmed lung disease associated with IgG4-RD.

## Methods

This SLR was conducted in accordance with the Cochrane Handbook and reported as per the Preferred Reporting Items for Systematic Reviews and Meta-Analyses guidelines [11, 12]. The protocol was developed by KK and MD and registered in the PROSPERO database of systematic reviews (CRD42023416410) [13]. The research question was: What are the pulmonary manifestations described in IgG4-RD?

The population was defined as adult patients (≥ 18 years old) with a clinician-confirmed diagnosis of IgG4-RD. The main primary outcome was pulmonary manifestations in patients with IgG4-RD, including (but not limited to): mediastinal lymphadenopathy; ground glass changes; pulmonary infiltrates; bronchiectasis; broncho-vascular bundle thickening; pleural thickening; pleural effusion; pulmonary fibrosis; pulmonary nodule; alveolar haemorrhage; consolidation and bronchial wall thickening. Secondary outcomes were treatment and prognosis.

### Search strategy, databases searched and study selection

The search strategy was developed by two authors (KK and MD) with the help of a librarian with expertise in undertaking clinical research and systematic reviews (HE) (MeSH terms are available in Supplementary file 1). Medline, Embase and Cochrane Databases were searched. Articles discussing IgG4-RD, published until February 2024 were included in this review.

Inclusion criteria were: articles discussing patients with clinician-diagnosed and biopsy-proven IgG4-RD, and articles published in English language. Exclusion criteria were cases of IgG4-RD without pulmonary manifestations, as well as single case reports, case series of less than five patients, opinion articles and reviews.

Titles and abstracts were screened by CD and CJ, to assess eligibility. The full articles which met the inclusion criteria were then examined in detail by CD and CJ, with 20% further examined by KK. In addition to basic demographics, information was extracted on type and prevalence of pulmonary manifestations, comorbidities, management and outcomes.

### Assessment of risk of bias, data extraction and synthesis

Risk of bias for each included study, where appropriate, was assessed using Newcastle–Ottawa Scale [14]. Data extraction from the included articles was undertaken by CD and CJ. No papers or additional data or online supplemental material were required from authors.

Demographic data of the study cohort were collected for each article, as well as number of reported cases with pulmonary involvement; subtypes of pulmonary manifestations; management options; clinical outcomes and summary of authors’ conclusions, where mentioned.

## Results

**Fig. 1:**
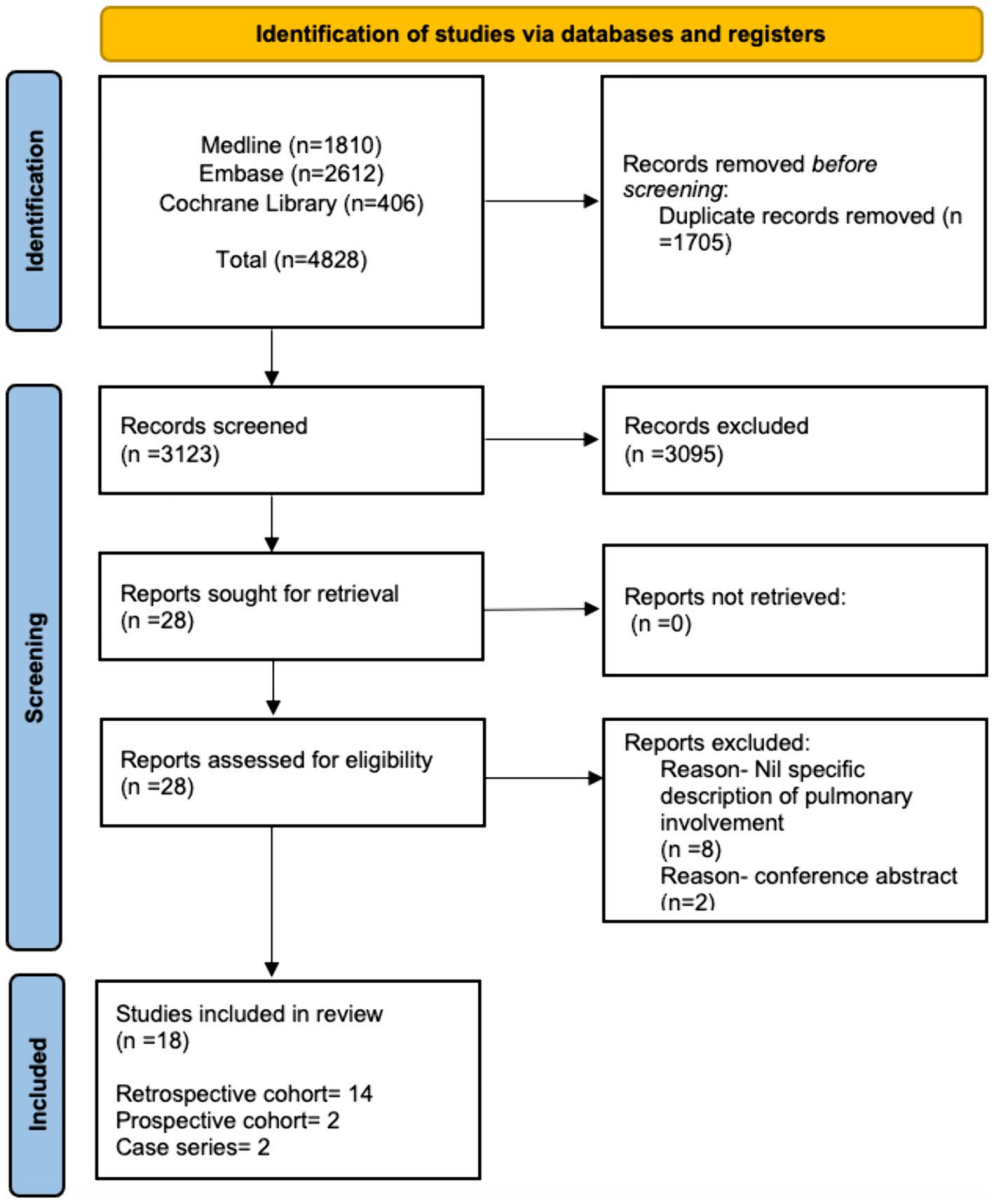
Flow diagram of stages of systematic literature review. Cochrane Library encompasses library of systematic reviews; systematic review protocols; controlled clinical trials

The initial search retrieved 3123 articles, after deduplication. Titles and abstracts were screened for eligibility, with ultimately 18 articles (14 retrospective cohort studies, two prospective cohort studies, two case series) included (Fig. 1). Included articles were deemed to be of low-medium quality as per risk of bias assessment (Table 1).

**Table 1:** Risk of bias (Newcastle–Ottawa Scale)

		Selection				Comparability	Outcome		
References	Type of study	Representativeness of the exposed cohort	Selection of the non-exposed cohort	Ascertainment of exposure	Demonstration that outcome of interest was not present at start of study	Comparability of cohorts on the basis of the design or analysis	Assessment of outcome	Was follow-up long enough for outcomes to occur	Adequacy of follow up of cohorts
Cao et al. 2017 [22]	Retrospective cohort	*	*	*	*			*	
Corcoran et al. 2017 [2]	Prospective cohort	*	*	*	*		*	*	*
Fei et al. 2015 [15]	Prospective cohort	*	*		*		*		*
Kang et al. 2020 [19]	Retrospective cohort						*		*
Kasashima et al. 2019 [24]	Retrospective cohort			*				*	
Liu et al. 2021 [21]	Retrospective cohort			*	*		*	*	*
Liu et al. 2022 [16]	Retrospective cohort	*		*	*		*		
Lv et al. 2017 [25]	Retrospective cohort			*			*		
Matsui et al. 2013 [3]	Retrospective cohort	*	*	*	*	*	*	*	*
Muller et al. 2021 [17]	Retrospective cohort	*		*	*	*	*		
Ogoshi et al. 2015 [7]	Retrospective cohort						*		
Otani et al. 2018 [27]	Retrospective cohort			*			*		
Saraya et al. 2017 [26]	Retrospective cohort	*	*	*	*	*	*		
Sun et al. 2016 [6]	Retrospective cohort			*			*		
Xie et al. 2020 [23]	Retrospective cohort	*		*		*		*	
Zen et al. 2009 [18]	Retrospective cohort						*		

### General demographics

Of a total of 724 patients with a diagnosis of IgG4-RD, 381 had pulmonary manifestations.

The mean age at the onset of the disease, for the total cohort, was 59.4 (SD 5.8) years and 68.6% were male.

Demographics and clinical information are summarised in Table 2. The geographic distribution of our sample included 15 cohorts from Asia (with a total of 617 cases; 85.2% of the total); two papers from Europe (with a total of 101 cases; 14%) and one study from the United States of America (USA) (6 cases; 0.8%).

**Table 2:** Summary of patient demographics and pulmonary manifestations

Article information			Size and demographics			Co-morbidities		Pulmonary involvement		Pulmonary manifestations											
References	Country of study	Type of study	Number of patients	Male (%)	Mean age at onset (years)	Co-morbidities	Smoking (%)	n	%	ML	PN/M	GGC	BVT	PBVT	BWT	PT	PE	PD	C	P	BRE
Cao et al. 2017 [22]	China	Retrospective observational	17	70.6	61	n/a	41.2	9	52.9	n/a	4	4	1	n/a	n/a	n/a	n/a	n/a	n/a	n/a	2
Chen et al. 2016 [4]	China	Case series	12	75	55	n/a	n/a	6	50	n/a	6	6	4	n/a	n/a	n/a	n/a	n/a	n/a	n/a	n/a
Corcoran et al. 2017 [2]	UK	Prospective cohort	53	77.4	62.4	n/a	34	22	41.5	19		3		n/a	n/a	1	3	n/a	2	n/a	n/a
Fei et al. 2015 [15]	China	Prospective cohort	248	50.4	54.2	Allergy, asthma	n/a	87	35.1	46	22	8	20	n/a	n/a	14	4	n/a	n/a	n/a	n/a
Kang et al. 2020 [19]	Korea	Retrospective observational	37	78.4	55.6	HTN, diabetes, stroke, TB sequelae, COPD	75.5	37	100	n/a	11	2	8	n/a	n/a	n/a	n/a	n/a	11	n/a	n/a
Kasashima et al. 2019 [24]	Japan	Retrospective observational	22	72.7	65	Asthma, COPD, chronic bronchitis, diabetes, renal failure, asbestos exposure, eosinophilic rhinitis	n/a	8	36.3	n/a	2	n/a	n/a	n/a	n/a	1	6	n/a	n/a	2	n/a
Keenan et al. 2016 [20]	US	Case series	6	83.3	55.2	Chronic pancreatitis, ulcerative colitis, autoimmune hepatitis, juvenile rheumatoid arthritis, latent TB	33.3	6	100	4	4	2	n/a	n/a	n/a	n/a	n/a	n/a	n/a	n/a	n/a
Liu et al. 2021 [21]	China	Retrospective observational	10	90	59.7	TB, COPD	60	10	100	7	8	3	5	n/a	n/a	n/a	n/a	n/a	1	n/a	4
Liu et al. 2022 [16]	China	Retrospective observational	12	42	59.4	Asthma, allergy	n/a	11	92	11	2	6	n/a	n/a	11	n/a	n/a	n/a	n/a	n/a	n/a
Lv et al. 2017 [25]	China	Retrospective observational	7	57.1	57	n/a	n/a	7	100	2	1	6	5	n/a	n/a	n/a	1	n/a	n/a	n/a	2
Matsui et al. 2013 [3]	Japan	Retrospective observational	18	78	62	Allergy, asthma, Sjogren syndrome	72.2	18	100	18	10	n/a	16	n/a	n/a	n/a	n/a	n/a	4	n/a	n/a
Muller et al. 2021 [17]	France	Retrospective observational	48	81	60	Atopy,	19	43	89	15	7	5	n/a	25	n/a	n/a	n/a	4	n/a	n/a	n/a
Ogoshi et al. 2015 [7]	Japan	Retrospective observational	35	68.5	67	Asthma, allergic conjunctivitis	n/a	14	40	13	3	5	5	n/a	n/a	n/a	n/a	n/a	2	n/a	n/a
Otani et al. 2018 [27]	Japan	Retrospective observational	20	54	n/a	n/a	n/a	20	100	n/a	1	n/a	8	n/a	n/a	n/a	n/a	n/a	n/a	n/a	n/a
Saraya et al. 2017 [26]	Japan	Retrospective observational	52	61.5	63	n/a	n/a	16	30.8	14	7	11	7	n/a	n/a	n/a	n/a	n/a	n/a	n/a	4
Sun et al. 2016 [6]	China	Retrospective observational	17	35.3	44.8	Allergy, Sjogren syndrome, TB, chronic gastritis, diabetes, COPD, asthma, vitiligo, psoriasis, HTN	23.5	17	100	5	4	6	2	n/a	n/a	2	n/a	n/a	5	n/a	n/a
Xie et al. 2020 [23]	China	Retrospective observational	89	78	n/a	n/a	47.2	50	56	32	50	21	n/a	n/a	n/a	9	4	n/a	1	n/a	n/a
Zen et al. 2009 [18]	Japan	Retrospective observational	21	81	69	Allergy, asthma	n/a	21	100	n/a	9	1	4	n/a	n/a	4	n/a	n/a	n/a	n/a	n/a

*n/a* not available, *HTN* hypertension, *TB* tuberculosis, *COPD* chronic obstructive pulmonary disease, *ML* mediastinal lymphadenopathy, *PN/M* pulmonary nodule/mass, *GGC* groundglass changes, *BVT* bronchovascular thickening, *PBVT* peribronchovascular thickening, *BWT* bronchial wall thickening, *PT* pleural thickening, *PE* Pleural effusion, *PD* pleural disease, *C* consolidation, *P* pneumonia, *BRE* bronchiectasis

Patient comorbidities were reported in 11 of the 18 papers. The most predominant comorbidity was obstructive lung disease (in the form of chronic obstructive pulmonary disease or asthma), followed by allergy or atopy [3, 6, 15–18], tuberculosis (active, latent or previous), autoimmune disorders (autoimmune pancreatitis, autoimmune hepatitis, juvenile rheumatoid arthritis, ulcerative colitis, Sjogren syndrome, vitiligo, psoriasis and chronic bronchitis [2, 3, 6, 15–24]. Smoking was the main risk factor mentioned in association with the presence of comorbidities. Although not all papers gave explicit information regarding the proportion of smokers, from the available data 45.1% of the total cohort of patients were active or previous smokers.

### Pulmonary involvement and manifestations

Of the total number of patients, 381 (52.6%) had confirmed pulmonary involvement with tissue proven IgG4-RD. Where mentioned, the most common site used for biopsy was lung (*n* = 150), followed by extra-pulmonary site (non-specified) (*n* = 77) and lymph node biopsy (*n* = 11), comprising submandibular (*n* = 8), sub-clavicular (*n* = 2), inguinal (*n* = 1), and lacrimal gland (*n* = 1) (with some patients undergoing biopsy at more than one site).

Mediastinal lymphadenopathy was the most common feature, noted in 48.8% (*n* = 186) patients [2, 3, 6, 7, 15–18, 20, 21, 23, 25, 26]. Pulmonary nodules or masses were present in 39.6% of cases (*n* = 151), broncho-vascular thickening in 22.3% (*n* = 85) and ground glass changes in 23.4% (*n* = 89) [2–4, 6, 7, 15–27]. “Peri-broncho-vascular thickening” was described in 6.6% (*n* = 25), “bronchial wall thickening” in 3.7% (*n* = 14) and “thickening of the interlobular septa” in 2.4% (*n* = 9) [16, 17, 21, 23, 25].

Pleural involvement was described, with “*pleural thickening*” stated in 9.2% (*n* = 35), “*pleural effusion*” in 4.7% (*n* = 18) and “*pleural disease*” in 1% (*n* = 4) [2, 15, 17, 18, 21, 23–26]. Interstitial lung disease (ILD) was reported in 9.4% (*n* = 36), non-specific interstitial pneumonitis (NSIP) in eight patients and one patient was found to have alveolar haemorrhage [4, 6, 15, 17, 20, 23]

Other notable findings were of pulmonary consolidation (*n* = 26, 6.5%), pneumonia (*n* = 2), bronchiectasis (*n* = 14, 2.9%), alveolar interstitial involvement (*n* = 11, 2.6%) and large airway disease in the form of airway stenosis (*n* = 1) [2, 3, 6, 7, 18, 19, 21–27].

### Treatment of patients with IgG4-RD with pulmonary involvement

Data on treatment was available in 226 patients with IgG4-RD with pulmonary involvement, across 13 of the 18 studies included (Table 3). Two hundred eleven (93.4%) patients received glucocorticoids (GC), of which 93 (44.1%) had a combination of GC and at least one other immune-modulatory drug [2, 3, 6, 7, 15–24]. This included 31 patients with cyclophosphamide (CYC); 18 with azathioprine (AZA); six with mycophenolate mofetil (MMF); six with rituximab; five with methotrexate (MTX); and 50 with unspecified immunotherapy [2, 3, 6, 7, 15–21, 23, 24]. Twenty patients had surgical resection of the pulmonary nodule and one patient had a liver transplant [2, 18, 20, 21, 23]. Three of the patients had treatment with *Trypteryfium wilfordii*—a plant used in traditional Chinese medicine, in addition to glucocorticoids and other immunosuppressants [16, 28].

**Table 3:** Summary of treatment and clinical outcomes

	No. patients		Treatment		Immunosuppressant (if specified)					Other Tx	Clinical outcomes						
References	Total study	Had Tx	GC alone	GC + IS	Aza	MTX	Ritux	MMF	CYC		Remission (unspecified)	Complete remission	Partial remission	Relapse	Stable	Progression	Death
Corcoran et al. 2017 [2]	53	4	1	3	n/a	2	n/a	1	n/a	1 SR							7
Fei et al. 2015 [15]	79	72	23	48	n/a	n/a	1	n/a	n/a		64				20		
Kang et al. 2020 [19]	37	31	16	15	15	n/a	n/a	n/a	n/a			2	16	6	6	2	
Kasashima et al. 2019 [24]	8	3	2	1	n/a	n/a	n/a	n/a	n/a			2				1	
Keenan J 2016	6	5	3	2	n/a	n/a	n/a	2	n/a	1 LT	1			3		1	
Liu et al. 2021 [21]	10	10	5	2	n/a	n/a	n/a	n/a	2	2 SR, 1 ST	6			2	2		
Liu et al. 2022 [16]	12	12	4	8	n/a	n/a	n/a	n/a	8	3 TW	12						
Matsui et al. 2013 [3]	17	15	15	n/a	n/a	n/a	n/a	n/a	n/a		15						
Muller et al. 2021 [17]	48	8	2	6	1	n/a	5	n/a	n/a			2	4		2		
Ogoshi et al. 2015 [7]	14	13	13	n/a	n/a	n/a	n/a	n/a	n/a		9			5			
Sun et al. 2016 [6]	17	14	8	6	1	n/a	n/a	1	4					4	9		1
Xie et al. 2020 [23]	50	23	n/a	23	1	3	n/a	2	17	6 SR			48				
Zen et al. 2009 [18]	21	18	7	n/a	n/a	n/a	n/a	n/a	n/a	11 SR		12	3		2		
Total	372	226	130	93	18	5	6	6	31	25	107	18	71	20	35	4	8

*Tx* treatment, *n/a* not available, *GC* glucocorticoids, *IS* immunosuppressant, *Aza* azathioprine, *MTX* methotrexate, *Ritux* rituximab, *MMF* mycophenolate mofetil, *CYC* cyclophosphamide, *SR* surgical resection, *LT* liver transplant, *ST* symptomatic treatment, *TW* tripterygium wilfordii

Prednisolone was the commonest glucocorticoid given for treatment at a mean dose of 40mg/day (ranging from 15 to 60 mg). Duration of treatment ranged between 14 and 24 months [2, 3, 6, 7, 15–21, 23, 24].

### Clinical outcomes and prognosis

Clinical outcomes were reported in 263 patients among the 13 studies [2, 3, 6, 7, 15–21, 23, 24]. A total of 196 patients had remission of their disease, 20 patients had relapsing disease, 35 had stable disease, four had progressive disease and eight patients died due to pulmonary complications of IgG4-RD. Of the patients who had remission, 18 were reported to have complete remission; 71 patients had partial remission; and 107 had unspecified remission (i.e., not reported as complete or partial) [2, 3, 6, 7, 15–21, 23, 24].

## Discussion

In this SLR, we have demonstrated that pulmonary manifestations are common in patients with IgG4-RD, with over 50% of patients in our pooled cohort having one or more forms of pulmonary involvement. Included studies were deemed to be of low-medium quality, and there was marked heterogeneity in terms of reporting demographics, clinical aspects and treatment.

### Overview of demographics

Most studies included in this SLR were conducted in Asia, the majority in China. Studies identified from other regions were limited, with only one study (including six patients) from North America and two studies from Europe. Furthermore, specific information about the patient’s ethnicities was not available in the articles. Therefore, we are unable to draw any potential associations between patients’ ethnicities and the incidence of IgG4-RD based on this study alone.

Our total cohort of 724 patients with IgG4-RD comprised predominately male patients, with a male to female ratio of 2.2:1. Obstructive lung disease (either in the form of asthma or COPD) was the most frequently described comorbidity. It is important to note, given the presence of such respiratory comorbidities, it is unclear how much of the patients’ pulmonary involvement, is due to IgG4-RD or existing lung pathology in such cases. Nevertheless, with regards to pulmonary comorbidities specifically, a small retrospective observational study performed in China in patients with new adult-onset asthma, recommended investigations for IgG4-RD in patients who present with hyper-eosinophilia, high IgE and IgG levels [16]. While this could potentially bring an additional safety net for misdiagnosing patients with IgG4-RD with adult-onset asthma, it has the potential to miss those with IgG4-RD who do not have raised serum IgG4 levels.

Where reported, 41.5% of our cohort were smokers. While smoking is a recognised risk factor in development of obstructive lung disease, it remains unclear whether there is an association between smoking and lung involvement in IgG4-RD [29].

As mentioned previously, other autoimmune conditions have been reported in association, or alongside IgG4-RD. Notably, we identified a case of autoimmune hepatitis that required liver transplantation. In this case, it was observed that the pulmonary manifestations resolved after the transplantation [20]. It is possible that the resolution may have been secondary to immunosuppression following liver transplantation.

### Pulmonary manifestations

The largest cohort study included in our review, 248 patients with IgG4-RD, reported involvement of almost all intrathoracic organs [15]. One observational study in Japan, classified the pulmonary changes in IgG4-RD in four categories: solid nodular, broncho-vascular, alveolar interstitial and ground-glass round opacity [18]. However, this classification does not seem to be comprehensive enough for all manifestations as it does not include, for example, lymphadenopathies or pleural disease. The most common feature identified in our SLR, alone or in combination of other pulmonary features, was the presence of mediastinal lymphadenopathies in 48.8% (*n* = 186).

The second most common feature was represented by pulmonary nodules or masses (*n* = 89), followed by broncho-vascular thickening (*n* = 85). When using the original terminology in the retrieved articles, we identified “*peribronchovascular thickening*” described in 25 patients, “*bronchial wall thickening*” in 14 patients and “*thickening of the interlobular septa*” in nine patients. If these findings were to be interpreted as “broncho-vascular changes” this would comprise 133 patients, making it the second most common feature [17, 21, 23, 25]. Given the overall lack of guidance on categorising and describing pulmonary manifestations in IgG4-RD, this is a good point of reflection regarding the need for consistency when describing or reporting clinical findings.

As stated above, pleural disease appears to be another significant manifestation in IgG4-RD. We have identified that 14.9% of the patients had some type of pleural involvement in the form of pleural effusions, pleural thickening or pleural disease. Kasashima et al. concluded in 2009 that the most common feature of pleural involvement in IgG4-RD is pleural effusions, and that histologically, this can be characterised by high infiltration of eosinophils and IgG4 + cells [24]. Therefore, more detailed diagnostic investigations for pleural manifestations of unclear aetiology might point towards a diagnosis of IgG4-RD.

Despite IgG4-RD being described as a fibrosing and inflammatory disease, ILD was stated as a definitive diagnosis in a minority of the patients (only 9.4%, *n* = 36) with 8 cases of nonspecific interstitial pneumonia (NSIP) and one case of alveolar haemorrhage in a patient with a positive ANCA [6, 20]. It is difficult to interpret if there is an overlap between ILD or vasculitis and IgG4-RD. More data is needed in this regard to draw any conclusions. However, it is worth considering how many patients with steroid-responsive ILD (or vasculitis with immunosuppression) could potentially have a diagnosis of IgG4-RD.

Pulmonary consolidation or pneumonia was described as an important finding in IgG4-RD by Sun in 2016. Given the high incidence of general medical patients requiring hospital admission, who are found to have lung consolidations/pneumonia, this statement is extremely relevant to clinical practice. Pneumonias that do not resolve despite adequate treatment should be investigated and IgG4-RD should be considered as a differential diagnosis for these cases.

### Treatment strategies and clinical outcomes

The majority of patients received GC treatment, sometimes in conjunction with immune-modulatory therapy. In studies where treatment strategies were reported, patients receiving treatment showed greater clinical improvement compared to those who did not receive treatment or those with varied compliance to treatment [20, 24]. This was evidenced by reduced IgG4 levels as well as decreased lymphadenopathy and pulmonary nodule size on follow up CT scan [20, 21].

The optimal dose and course of GC for IgG4-related lung disease remains unclear, and this varied across the included studies. In one cohort, 72 out of 79 patients received GC therapy, with a median initial dose of prednisone of 40mg per day, out of which 48 patients received combination therapy with additional immune-modulation [15]. Intrathoracic lesions improved by 30% in 64 out of 72 patients (88.9%). Low dose of prednisone (< 10 mg/day) with or without other immune-modulation was used as maintenance therapy to achieve remission [15].

In patients who had a tapering course of prednisolone, for example, a reduced dose of 10-20mg per day, had a higher risk of relapse and required more intensive treatment to manage their disease [16]. Additional immune-modulating agents such as CYC, MMF or AZA were introduced among patients with evidence of relapsing disease following unsuccessful treatment with prednisolone alone [2, 15]. Reintroduction of GC in combination with additional immunosuppressive medication led to a reported improvement in the patients who experienced relapse [21]. Few studies reported that remission was achieved in all patients after intensive treatment with increasing doses of oral prednisolone and/or combined therapy with an additional immunosuppressive agent such as CYC, evidenced by shrinkage of pulmonary nodules and broncho-vascular thickening on follow-up CT scan [16, 21]. Rituximab was used as third-line treatment agent in patients with multiorgan involvement who were resistant to treatment with GC and other immune-modulating therapy [2, 15]. Where intrathoracic lesions improved after treatment with GC, low-dose prednisone, with or without immune-modulation, was used as maintenance therapy and stopped after achieving stable disease after a few years [15, 20].

Overall, these results are in favour of initial aggressive therapy to reduce the likelihood of requiring further therapeutic agents later in the disease course. Although long-term use of GC are known to cause adverse effects such as weight gain, skin atrophy, osteoporosis, diabetes, and increased susceptibility to infection, higher doses in the initial phase appear to reduce the likelihood of relapse and further GC [30]. Moreover, intensive treatment with higher prednisolone dose of 40mg or above at the initial stage reduces the overall duration and amount of GC use overall, as well as being associated with disease remission in the long term [16].

Patients who did not receive any form of treatment were reported to have persistent pleural effusion, progressive increase in the volume of the effusion and eosinophilic pneumonia resulting in gradual deterioration of respiratory function [24]. A lower rate of treatment response was observed in patients presenting with storiform fibrosis [17]. Patients who were refractory to treatment showed advanced stage of pulmonary disease associated with IgG4-RD, with greater degree of fibrosis affecting the pulmonary tissues [24]. Therefore, early treatment is important to decrease the likelihood of the development of these histological changes at diagnosis, irreversible organ damage and progression to advanced disease [24].

In general, the overall prognosis is poor in advanced pulmonary fibrosis and the scope of treatment diminishes as the disease progresses. For example, progressive pulmonary fibrosis leads to respiratory failure within 2–5 years of diagnosis in most instances [31]. Therefore, based on the findings from our pooled cohort, it would appear that investigations for pulmonary disease in IgG4-RD must be done in the early stages in patients presenting with idiopathic pleural lesions and pulmonary disease [24]. This may facilitate early diagnosis and initiation of treatment, prevent progression into irreversible pulmonary fibrosis, and reduce the morbidity and mortality associated with pulmonary manifestations in IgG4-RD.

Although early treatment reduces progression of disease, the duration of treatment appears to be guided by the patients’ symptoms and extent of disease on presentation [15, 20]. For example, patients who had asymptomatic, incidental presentation of lung nodules with stable appearances on follow up CT scan had monitoring with spirometry, and remained stable without treatment [20]. Overall, there appears to be a lack of data in assessing clinical outcomes among patients with different extents of disease. There is a need for future studies to classify, monitor and follow up clinical outcomes in patients with different stages of disease. Assessing progression and remission among patients would help to guide treatment decisions in patients with asymptomatic or stable disease on presentation, as well as those with more advanced pulmonary involvement, which carries high morbidity.

### Strengths and limitations

This systematic review summarises the pulmonary manifestations, treatments, and outcomes in patients with IgG4-RD. IgG4-RD is a rare disease. Despite this, our review comprised a cohort of over 380 patients with pulmonary manifestations in IgG4-RD. It is not possible, from our study, to definitively conclude, however, if these manifestations are truly associated with IgG4-RD only.

It is important to note that the studies included in this review were of low-medium quality, therefore this limits generalisation of results. The ACR/EULAR diagnostic criteria were published in 2019, which has likely led to more rigorous assessment and diagnosis of patients presenting with potential symptoms of IgG4-RD. Some of the papers included in our study were published prior to the development of these diagnostic criteria, but there may be under-reporting of cases prior to 2019, due to a lack of diagnostic criteria. Furthermore, the articles included in our study were markedly heterogenous, in terms of the data presented as well as duration of, and investigations performed during, follow-up. Most articles were from Asian countries, with two papers from Europe and one from North America. Therefore, the results presented should be interpreted with caution in terms of generalisability across populations. In the larger cohort studies, only pooled demographic data were available on IgG4-RD, and not those with pulmonary manifestations specifically. Few studies reported data on follow up such as imaging and pulmonary function tests, and only 13 of the 18 studies reporting pulmonary manifestations described treatment strategies. In some studies, clinical outcomes were reported without differentiating patients who received treatment and those who did not, making direct comparisons difficult.

## Conclusion

In conclusion, pulmonary manifestations are common in patients with IgG4-RD. Over half of the patients included in our SLR with a confirmed diagnosis of IgG4-RD had respiratory involvement. The lung manifestations can be varied and heterogenous, but the most common feature was mediastinal lymphadenopathy. Glucocorticoids remain the mainstay of treatment with good overall response. However, other immunosuppressive agents can be used in cases resistant to steroid therapy.

Our SLR is intended to be a reflection point for clinicians and encourage further studies to characterise pulmonary manifestations in IgG4-RD and their treatment. Active consideration of IgG4-RD is needed in patients who present with pulmonary disease, especially if unexplained and in the presence of systemic involvement, in order to reduce the morbidity and mortality resulting from this condition.

## Supplementary Information

Below is the link to the electronic supplementary material.

Supplementary file1 (DOCX 32 KB)

Supplementary file2 (DOCX 34 KB)

Supplementary file3 (DOCX 14 KB)

Supplementary file4 (DOCX 14 KB)

## Data Availability

All data available upon reasonable request.
