# Supplementary material for: Pulmonary manifestations, treatments and outcomes of IgG4-related disease–a systematic literature review
Source: Rheumatol Int. 2024 May 20;44(10):1875–86. doi: 10.1007/s00296-024-05611-7 (PMC11393110; doi:10.1007/s00296-024-05611-7)
Supplement: Supplementary file 3 — Supplementary file3 (DOCX 14 KB) [file 296_2024_5611_MOESM3_ESM.docx]

| **Patient/Population/Problem** | **Intervention** | **Comparison** | **Outcome** |
| --- | --- | --- | --- |
| (Immunoglobulin G4 related or Immunoglobulin G4 associated) adj2 disease$  or  IgG4$1 or "IgG 4" | N/A | N/A | lung diseases or bronchiolitis obliterans or bronchiectasis or pleural effusion or idiopathic interstitial pneumonias or pulmonary embolism  Or  pulm$ or lung$ or ILD or respir$ or pneum$ or pleur$ or COPD or NSIP or airway$ or bronchiolitis or bronchiectasis or alveolar h?emorrhage |

**Supplementary material: MeSH terms used in search strategy (PICO elements)**
